# Undifferentiated Intimal Sarcoma of the Large Abdominal Arteries Presenting As Acute Mesenteric Ischemia: A Case Report

**DOI:** 10.7759/cureus.107091

**Published:** 2026-04-15

**Authors:** Christian Benignus, Steffen Retter, Abbas Agaimy, Johannes Gahlen, Thomas Schiedeck

**Affiliations:** 1 General Surgery, Hospital Ludwigsburg (RKH), Ludwigsburg, DEU; 2 General Surgery, Private Medical University Salzburg, Salzburg, AUT; 3 Pathology, Friedrich-Alexander-University Hospital Erlangen-Nürnberg, Erlangen, DEU; 4 Vascular and Endovascular Surgery, Hospital Ludwigsburg (RKH), Ludwigsburg, DEU

**Keywords:** acute arterial thrombosis, acute mesenteric ischemia (ami), aorta surgery, intimal sarcoma, malignant vascular tumor, visceral artery occlusion

## Abstract

Primary malignant tumors of the large arteries are exceedingly rare and diagnostically challenging. Undifferentiated intimal sarcomas and leiomyosarcomas are the major types encountered. Intimal sarcomas are highly aggressive neoplasms characterized by intraluminal growth and a tendency to mimic thromboembolic disease, often resulting in delayed diagnosis and poor outcomes. We report the case of a 63-year-old man who presented with progressive abdominal pain and weight loss and rapidly developed acute mesenteric ischemia due to extensive intraluminal obstruction of the descending and abdominal aorta with involvement of major visceral branches. Despite emergency surgical interventions, including thrombectomy, vascular bypass, and four re-laparotomies, the clinical course was marked by rapid re-thrombosis, sepsis, and multiorgan failure. Histopathological examination of retrieved intravascular material revealed a high-grade undifferentiated sarcoma that was confirmed at autopsy as an undifferentiated intimal sarcoma of the large arteries. This case highlights the diagnostic pitfalls of malignant vascular tumors and underscores the importance of considering intimal sarcoma in patients with atypical or recurrent arterial thrombosis, particularly when multiple visceral vessels are involved.

## Introduction

Primary malignant tumors of large arteries are exceedingly rare and represent a diagnostically and therapeutically challenging entity [1]. Among these, intimal sarcomas are highly aggressive mesenchymal neoplasms arising from the intimal layer of large vessels, the innermost layer of the vascular wall, most frequently involving the aorta or pulmonary artery, although any large vessel may rarely be affected [2]. Despite advances in imaging and vascular surgery, prognosis remains poor, largely due to delayed diagnosis and rapid disease dissemination [3].

Clinically, intimal sarcomas often present with nonspecific symptoms, including weight loss, abdominal pain, or ischemic events. Their predominant intraluminal growth pattern frequently leads to misinterpretation as thromboembolic disease or advanced atherosclerosis. As a result, diagnosis is commonly established at an advanced stage, often during emergency surgery or postmortem examination [4].

Radiological differentiation between malignant vascular tumors and benign thrombotic processes remains difficult, even with contrast-enhanced imaging. Histopathological and immunohistochemical analyses are therefore essential for definitive diagnosis, allowing exclusion of important differential diagnoses such as sarcomatoid carcinoma, metastatic malignancy, or primary thrombotic disease.

We present a rare case of undifferentiated intimal sarcoma of the large arteries manifesting as acute mesenteric ischemia with rapid clinical deterioration. We present this case to highlight the diagnostic challenges of this entity and to emphasize the importance of considering malignant vascular tumors in patients with atypical or recurrent arterial thrombosis.

## Case presentation

A 63-year-old man presented to the emergency department with progressive abdominal pain over a period of ten days. Over the preceding months, he reported an unintentional weight loss of approximately 10 kg. His medical history was notable for arterial hypertension, type 2 diabetes mellitus, and multiple coronary artery bypass graft surgeries. He was a non-smoker, reported occasional alcohol consumption, and had no known family history of malignancies. At the time of admission, his regular medications included acetylsalicylic acid, atorvastatin, bisoprolol, metformin, ezetimibe, and cholecalciferol.

Baseline laboratory parameters are summarized in Table 1. In addition to elevated inflammatory markers, procalcitonin, an early marker of sepsis, was mildly increased on admission.

**Table 1:** Initial blood parameters at first presentation. CRP: C-reactive protein; INR: international normalized ratio.

Parameter	Result	Reference Range
Leukocytes	20.1 × 10³/µL	4.3-10 × 10^3^/µl
Hemoglobin	13.6 g/dl	14.0-17.5 g/dl
Platelet count	412 × 10^3^/µl	150-400 × 10^3^/µl
INR	1.36	0.7-1.3
Sodium	134 mmol/l	135-145 mmol/l
Potassium	3.4 mmol/l	3.5-5.1 mmol/l
Serum lactate	1.7 mmol/l	0.56-1.39 mmol/l
Total bilirubin	1.3 mg/dl	<1.3 mg/dl
Creatinine	0.62 mg/dl	0.7-1.2 mg/dl
CRP	64.4 mg/l	<5 mg/l
Interleukin-6	110 pg/ml	<7 pg/ml
Procalcitonin	0.11 ng/ml	<0.05 ng/ml

**Figure 1:**
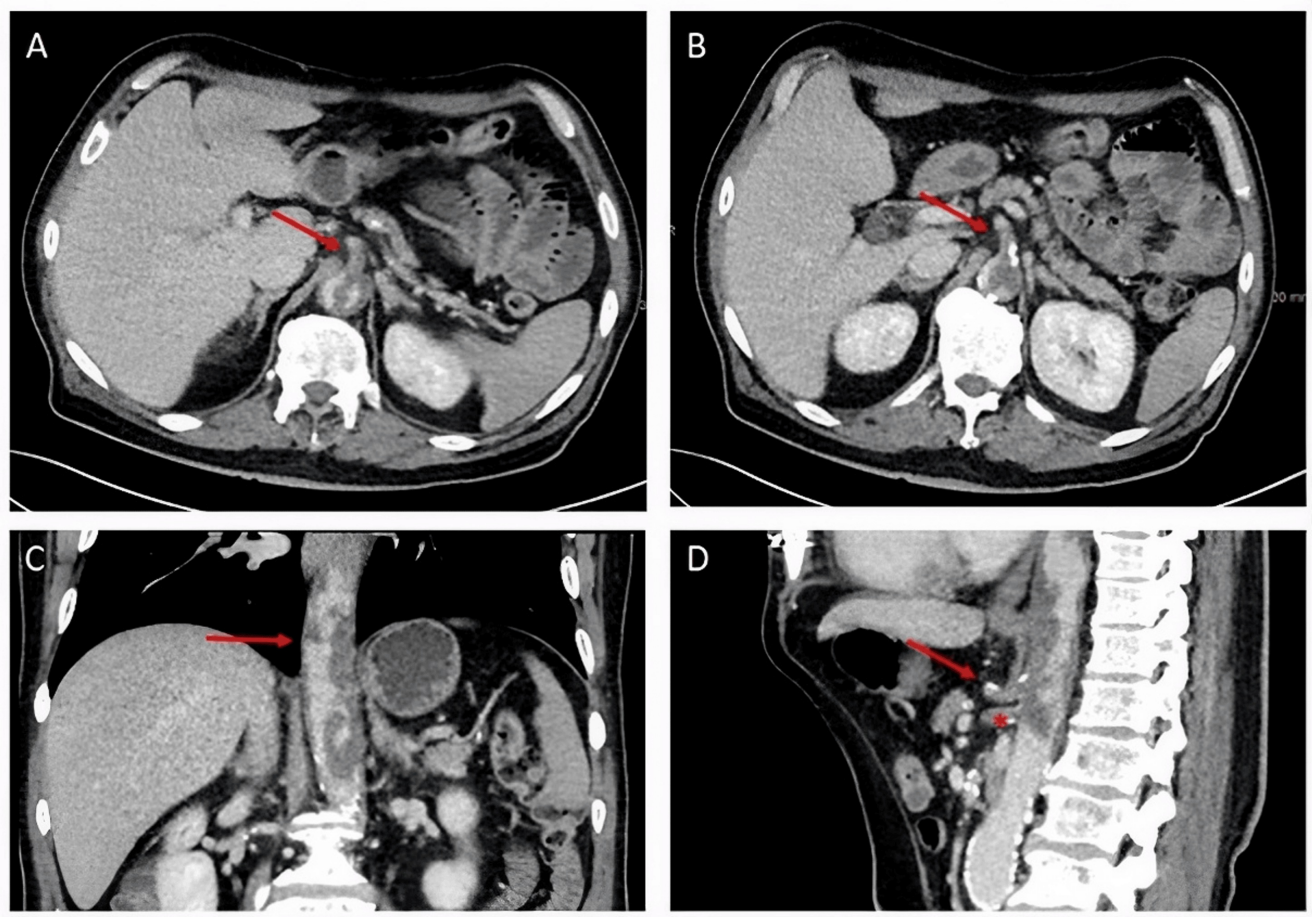
Contrast-enhanced CT imaging demonstrating thrombosis of the superior mesenteric artery (SMA), the celiac trunk, and the descending and abdominal aorta. A: Axial contrast-enhanced CT scan showing thrombosis of the SMA (arrow); B: Axial contrast-enhanced CT scan showing thrombosis of the celiac trunk (arrow); C: Coronal contrast-enhanced CT scan demonstrating thrombosis of the descending and abdominal aorta (arrow); D: Sagittal contrast-enhanced CT scan showing thrombosis of the celiac trunk (arrow) and the SMA (asterisk). CT: computed tomography; SMA: superior mesenteric artery.

Contrast-enhanced computed tomography (CT) demonstrated a band-like thrombotic occlusion of the descending and abdominal aorta, with complete occlusion of the celiac trunk and superior mesenteric artery (SMA) (Figure 1). These findings resulted in hypoperfusion of multiple abdominal organs and signs of impaired intestinal transit. Additionally, multiple hypodense lesions were identified in the spleen and liver. Thoracic CT showed no evidence of pulmonary metastases or mediastinal lymphadenopathy.

**Figure 2:**
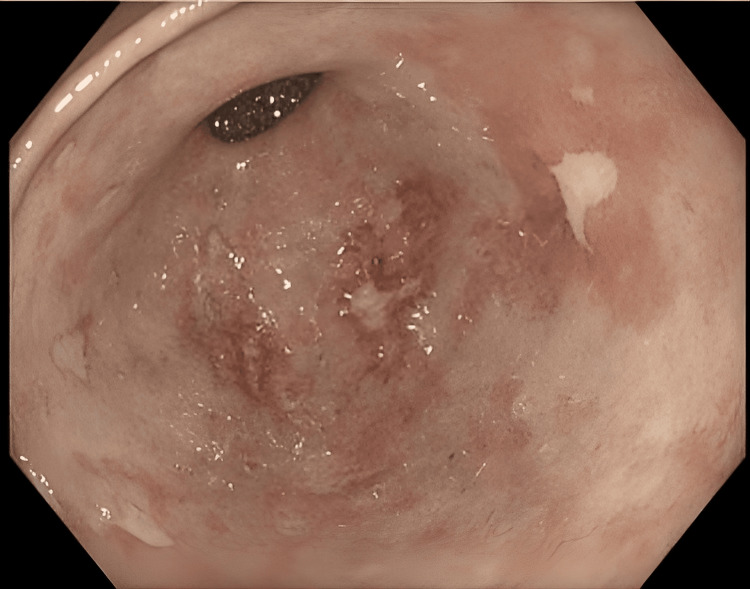
Gastroscopic view demonstrating multiple ischemic ulcers in the gastric antrum.

Esophagogastroduodenoscopy revealed multiple ischemic gastric ulcers (Figure 2). Ileocolonoscopy demonstrated nonspecific chronic inflammatory changes, which were interpreted as secondary to intestinal hypoperfusion.

During hospitalization, the patient developed septic shock with rapidly rising serum lactate levels, prompting emergency exploratory laparotomy. Intraoperatively, the stomach and colon appeared viable, while the small intestine was diffusely cyanotic but showed no signs of irreversible ischemia. The celiac trunk and SMA were exposed via the omental bursa, followed by thrombectomy of both vessels. Adequate inflow was restored, resulting in improved intestinal perfusion. Thrombotic material was collected and submitted for histopathological examination.

**Figure 3:**
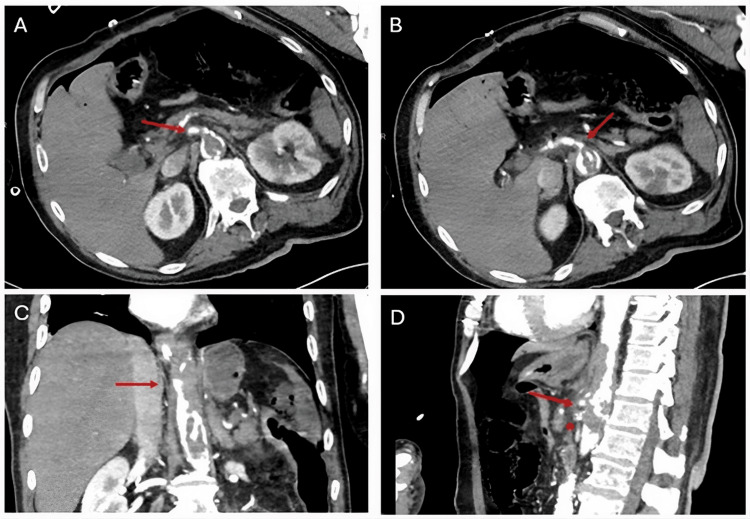
Four hours postoperatively, repeat contrast-enhanced CT imaging revealed recurrent thrombosis of both the SMA and the celiac trunk. A: Axial contrast-enhanced CT scan showing thrombosis of the SMA (arrow); B: Axial contrast-enhanced CT scan demonstrating thrombosis of the celiac trunk (arrow); C: Coronal contrast-enhanced CT scan illustrating involvement of both vessels (SMA, arrow; celiac trunk, asterisk); D: Sagittal contrast-enhanced CT scan confirming thrombosis of the celiac trunk (arrow) and the SMA (asterisk). CT: computed tomography; SMA: superior mesenteric artery.

Four hours postoperatively, repeat CT imaging revealed recurrent thrombosis of both vessels as well as newly developed hypoperfusion of the left kidney (Figure 3). An emergent re-laparotomy was performed, during which a bypass from the left internal iliac artery to the SMA was created using a 6-mm ring-reinforced silver graft soaked in rifampin. Due to extensive ischemic injury, approximately 3 meters of jejunum and 1 meter of ileum were resected. The bowel ends were left in discontinuity, and the abdomen was temporarily closed using negative-pressure wound therapy in accordance with a damage control surgery concept.

Three days later, a third-look laparotomy was performed, during which additional jejunal resection and formation of an end jejunostomy were required. Another three days later, a fourth-look laparotomy followed, including further jejunal wedge resections and an atypical (non-anatomical wedge) resection of liver segment III, as a primary hepatic malignancy was suspected after multidisciplinary consultation. Restoration of intestinal continuity was not feasible. In total, approximately 440 cm of small intestine were resected across all procedures, leaving a remaining length of approximately 60 cm.

**Figure 4:**
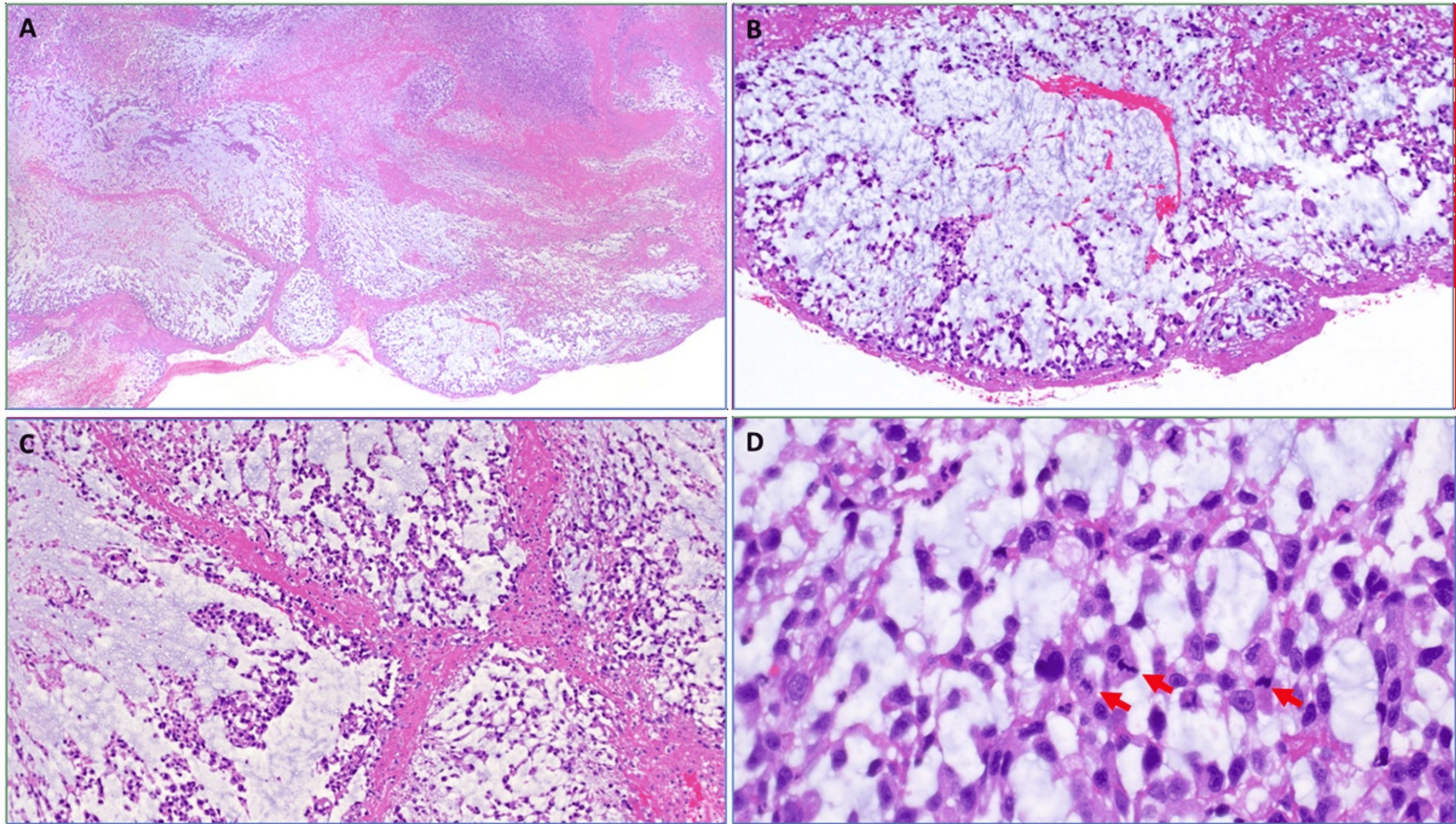
Histopathological analysis. A: low power shows large lobulated myxoid mass covered by fibrinous cap (thrombus-like) with extensive necrosis (HE, 50x); B: high power view of the smooth surface indicating intravascular growth (HE, 100x); C: prominent tumor lobules within myxoid background bordered by eosinophilic fibrinous bands (HE, 100x); D: high-power showing significant pleomorphism of tumor cells and brisk mitotic activity (arrows, HE, 200x). HE: hematoxylin and eosin stain.

Histopathological analysis (Figure 4) of the thrombotic material revealed a high-grade undifferentiated sarcoma with prominent myxoid features (myxofibrosarcoma-like) and variable expression of low molecular weight keratin and carbonic anhydrase IX, but otherwise lacking a specific line of differentiation by immunohistochemistry. It was negative for S100, Melan-A, cluster of differentiation (CD)61, CD34, desmin, multiple myeloma 1 (MUM1), smooth muscle actin, ERG, CD30, epithelial membrane antigen, CD117, paired box 8 (PAX8), CDK4, mouse double minute 2 homolog (MDM2), GATA3, and anaplastic lymphoma kinase (ALK), with preserved expression of SWItch/sucrose non-fermentable chromatin remodeling complex (SWI/SNF) complex proteins ARID1A, SMARCB1, SMARCA4, and PBRM1. The liver specimen demonstrated ischemic changes only, without evidence of malignancy. Differential diagnoses included metastatic sarcomatoid carcinoma (including renal cell carcinoma) and mesothelioma.

Given the non-resectable extent and highly aggressive nature of the disease, the therapeutic strategy was shifted to palliative care. This decision was made in agreement with the patient and his family after thorough interdisciplinary discussion. The patient received symptom-directed treatment, including adequate analgesia and intravenous fluid support, as oral intake was not possible. He was extubated, transferred to the general ward, and maintained on parenteral nutrition. He died four days later.

Autopsy revealed a tumorous lesion within the descending aorta above the renal arteries, extending into the mesenteric vessels and intermingled with thrombotic material. The intimal layers of the aortic wall were markedly thickened. Secondary massive dilation of the left ventricle was observed. No tumor involvement was identified in other organs, including the kidneys, renal vessels, lungs, pulmonary vessels, and the heart. The cause of death was determined to be septic multiorgan failure secondary to severe intestinal ischemia caused by the vascular tumor.

Based on the combined clinical, radiological, histopathological, and autopsy findings, the final diagnosis was undifferentiated intimal sarcoma of the large arteries, a rare and highly aggressive malignancy characterized by intraluminal growth, early vascular occlusion, rapid clinical deterioration, and poor prognosis.

## Discussion

Primary malignant tumors of the aorta are exceptionally rare, yet they are associated with substantial morbidity and mortality because diagnosis is frequently delayed and curative therapy is often no longer feasible at presentation. Intimal sarcoma, a high-grade mesenchymal neoplasm arising from the intimal layer of large vessels, is among the most aggressive entities in this group and typically exhibits predominant intraluminal growth with superimposed thrombosis and distal embolization. Large case series and pooled analyses consistently report short median survival. In a pooled analysis of 165 cases, Rusthoven et al. reported a median survival of 11 months, with one-, three-, and five-year survival rates of 46.7%, 17.1%, and 8.8%, respectively [5]. Prognosis is particularly poor in patients with visceral aortic involvement and incomplete resection, while multimodal strategies (complete surgical resection when possible plus adjuvant therapy) appear to be associated with improved outcomes.

A major challenge is that intimal sarcoma often masquerades as thromboembolic disease, vasculitis, or advanced atherosclerosis [6,7]. Patients may present with nonspecific constitutional symptoms (e.g., weight loss, inflammatory markers) and ischemic manifestations that dominate the clinical picture as described in case-based reports [8]. This initial clinical impression of thrombotic disease can lead to repeated endovascular or surgical interventions for presumed thrombosis while the underlying malignancy remains unrecognized.

Our case illustrates this pitfall in an extreme form: band-like aortic apparent thrombosis with acute occlusion of the celiac trunk and SMA, rapid re-thrombosis after thrombectomy, and subsequent progression to catastrophic intestinal ischemia and septic multiorgan failure. Similar presentations have been described in the literature. Santonja et al. reported an aortic intimal angiosarcoma in a patient presenting with mesenteric ischemia and an apparent abdominal aortic thrombus, with the diagnosis confirmed by immunohistochemistry and autopsy [9]. Deiana et al. described infiltrating thoracic aortic angiosarcoma with a large floating thrombus causing acute mesenteric ischemia, further underscoring that malignant aortic tumors can clinically behave like embolic/thrombotic disease [10]. More recently, Wenkel et al. reported undifferentiated intimal sarcoma of the visceral aorta presenting with recurrent renovisceral ischemia and initial misclassification as inflammatory vasculopathy (Takayasu arteritis), highlighting how inflammatory phenotypes and vascular stenoses/occlusions can misdirect the diagnostic pathway [11]. In comparison to our case, these reports similarly demonstrate delayed diagnosis and initial misinterpretation as thrombotic or inflammatory disease; however, the present case is distinguished by the exceptionally rapid clinical deterioration with recurrent thrombosis and extensive bowel ischemia necessitating multiple surgical interventions.

Collectively, these reports align with the fulminant course observed in our patient and emphasize that rapidly recurrent arterial thrombosis, especially involving multiple visceral branches, should prompt consideration of malignant etiologies. This underlines the necessity of submitting thrombotic material removed at surgery to careful histopathological evaluation.

Contrast-enhanced CT is often the first-line modality in acute ischemic presentations, but distinguishing tumor from thrombus remains difficult. In published cases, suggestive features include a polypoid or lobulated intraluminal mass, atypical enhancement patterns, eccentric aortic wall thickening, pseudoaneurysm-like configurations, or persistent/recurring apparent thrombus despite appropriate therapy [12]. Our case demonstrated several of these features, including extensive intraluminal aortic involvement and rapid recurrence after thrombectomy, which may represent important radiological clues to an underlying malignant process.

These findings represent important clinical “red flags” for an underlying malignant process: the combination of extensive aortic involvement with simultaneous occlusion of the celiac trunk and SMA, coupled with rapid re-occlusion after technically adequate thrombectomy.

Histopathology is decisive, yet interpretation may be challenging because intimal sarcomas can display a variety of cytoarchitectural patterns and generally lack defining immunohistochemical markers, although expression of MDM2, CDK4, and variable keratin expression has been reported, which broadens the differential diagnosis to include visceral sarcomatoid carcinoma (if growing expansile and obliterating the underlying vessels) or metastatic sarcomatoid carcinoma and other metastatic sarcomas. In the present case, MDM2 and CDK4 were negative, further highlighting the heterogeneity of immunohistochemical findings and the associated diagnostic challenges. Cytokeratin expression has been reported in malignant aortic tumors, including intimal sarcoma/angiosarcoma, and can therefore represent a diagnostic trap if evaluated outside the clinical context [4].

In our case, the thrombus specimen showed a high-grade keratin-expressing malignancy; the extensive immunohistochemical work-up appropriately addressed key mimickers. The strong CA IX expression and focal renal cell carcinoma (RCC)-marker positivity raised the possibility of sarcomatoid renal cell carcinoma; however, autopsy excluded a renal primary and confirmed a primary intraluminal aortic tumor with extension into mesenteric vessels, establishing the diagnosis of undifferentiated intimal sarcoma of the large arteries.

Complete surgical resection with clear margins remains the mainstay of treatment and offers the best chance for prolonged survival; however, curative treatment is rarely achievable, and resection is frequently impossible because diagnosis is made late or the tumor involves critical aortic segments and branch vessels. In the largest surgical series and pooled analyses, incomplete resection and visceral aortic involvement are associated with poor outcomes, whereas chemotherapy and multimodal approaches have been linked to improved survival in selected patients [13].

Nevertheless, many patients present in extremis with acute ischemia, sepsis, or multiorgan failure - circumstances in which the priority necessarily shifts from oncologic control to life-saving revascularization and damage control surgery. In our patient, repeated thrombosis and extensive bowel ischemia precluded definitive oncologic management. The rapid deterioration, lack of resectability, and autopsy-proven intraluminal tumor spread into mesenteric vessels are consistent with the dismal prognosis described in prior reports.

## Conclusions

This case underscores the importance of considering malignant vascular tumors in patients presenting with atypical or recurrent arterial thrombosis involving multiple visceral vessels. Early histopathological evaluation of intravascular material should be pursued whenever feasible, particularly in cases of rapid re-thrombosis or unusual imaging findings. Clinicians should be aware of these potential “red flags” to avoid diagnostic delay and guide timely decision-making. Even with aggressive intervention, prognosis may remain unfavorable in cases complicated by fulminant mesenteric ischemia and sepsis. Further studies are needed to improve diagnostic strategies and to define optimal multimodal treatment approaches for this rare and aggressive disease.
